# Progress Toward Regional Measles Elimination — Worldwide, 2000–2021

**DOI:** 10.15585/mmwr.mm7147a1

**Published:** 2022-11-25

**Authors:** Anna A. Minta, Matt Ferrari, Sebastien Antoni, Allison Portnoy, Alyssa Sbarra, Brian Lambert, Sarah Hauryski, Cynthia Hatcher, Yoann Nedelec, Deblina Datta, Lee Lee Ho, Claudia Steulet, Marta Gacic-Dobo, Paul A. Rota, Mick N. Mulders, Anindya S. Bose, William A. Perea, Patrick O’Connor

**Affiliations:** ^1^Department of Immunization, Vaccines, and Biologicals, World Health Organization, Geneva, Switzerland; ^2^Center for Infectious Disease Dynamics, Pennsylvania State University, University Park, Pennsylvania; ^3^Center for Health Decision Science, Harvard T.H. Chan School of Public Health, Harvard University, Boston, Massachusetts; ^4^Department of Infectious Disease Epidemiology, London School of Hygiene & Tropical Medicine, London, United Kingdom; ^5^Global Immunization Division, Center for Global Health, CDC; ^6^Division of Viral Diseases, National Center for Immunization and Respiratory Diseases, CDC.

All six World Health Organization (WHO) regions have committed to eliminating measles.* The Immunization Agenda 2021–2030 (IA2030)^†^ aims to achieve the regional targets as a core indicator of impact and positions measles as the tracer of a health system’s ability to deliver essential childhood vaccines. IA2030 highlights the importance of ensuring rigorous measles surveillance systems to document immunity gaps and achieve 95% coverage with 2 timely doses of measles-containing vaccine (MCV) among children. This report describes progress toward measles elimination during 2000–2021 and updates a previous report (*1*). During 2000–2021, estimated global coverage with a first MCV dose (MCV1) increased from 72% to a peak of 86% in 2019, but decreased during the COVID-19 pandemic to 83% in 2020 and to 81% in 2021, the lowest MCV1 coverage recorded since 2008. All countries conducted measles surveillance, but only 47 (35%) of 135 countries reporting discarded cases^§^ achieved the sensitivity indicator target of two or more discarded cases per 100,000 population in 2021, indicating surveillance system underperformance in certain countries. Annual reported measles incidence decreased 88% during 2000–2016, from 145 to 18 cases per 1 million population, then rebounded to 120 in 2019 during a global resurgence (*2*), before declining to 21 in 2020 and to 17 in 2021. Large and disruptive outbreaks were reported in 22 countries. During 2000–2021, the annual number of estimated measles deaths decreased 83%, from 761,000 to 128,000; an estimated 56 million measles deaths were averted by vaccination. To regain progress and achieve regional measles elimination targets during and after the COVID-19 pandemic, accelerating targeted efforts is necessary to reach all children with 2 MCV doses while implementing robust surveillance and identifying and closing immunity gaps to prevent cases and outbreaks.

## Immunization Activities

WHO and UNICEF use data from 1) administrative coverage (calculated by dividing the number of vaccine doses administered by the estimated target population reported annually), 2) country estimates,^¶^ and 3) vaccination coverage surveys to estimate MCV1 and second dose MCV (MCV2) coverage through routine immunization services (i.e., not mass campaigns).** During 2000–2010, estimated MCV1 coverage increased worldwide from 72% to 84%. However, coverage stagnated at 84% to 86% during 2010–2019, decreased to 83% in 2020 during the COVID-19 pandemic, and further declined to 81% in 2021. Although regional variation exists, all six WHO regions reported a decline in MCV1 coverage since 2019, with only the European Region plateauing from 2020 to 2021 (Table 1).

**TABLE 1:** Estimates of regional immunization coverage with the first and second doses of measles-containing vaccine administered through routine immunization services, reported measles cases, and measles incidence, by World Health Organization region — worldwide, 2000–2021

Yr/WHO region (no. of countries in region)	Percentage				No. of reported measles cases^§^ (% of total cases)	Measles incidence^§,¶,^**
	MCV1* coverage	Countries with ≥90% MCV1 coverage^†^	MCV2* coverage	Reporting countries with <5 measles cases per 1 million population^§,¶^		
**Total (all regions)**						
**2000 (191)**	**72**	**44**	**17**	**33**	**853,479 (100)**	**145**
**2010 (193)**	**84**	**63**	**42**	**59**	**343,806 (100)**	**50**
**2016 (194)**	**85**	**61**	**67**	**64**	**132,490 (100)**	**18**
**2019 (194)**	**86**	**62**	**71**	**44**	**873,022 (100)**	**120**
**2020 (194)**	**83**	**51**	**72**	**57**	**159,073 (100)**	**21**
**2021 (194)**	**81**	**47**	**71**	**66**	**123,981 (100)**	**17**
**African**						
2000 (46)	53	9	5	6	520,102 (60.9)	832
2010 (46)	72	36	5	30	199,174 (57.9)	232
2016 (47)	68	34	22	49	36,269 (27.4)	37
2019 (47)	70	30	33	34	618,595 (70.9)	560
2020 (47)	69	19	40	30	115,369 (72.5)	106
2021 (47)	68	23	41	34	89,602 (72.3)	83
**Americas**						
2000 (35)	93	63	65	89	1,754 (0.2)	2
2010 (35)	93	74	67	100	247 (0.1)	0.3
2016 (35)	92	66	80	97	97 (0.1)	0.1
2019 (35)	87	69	72	89	21,971 (2.5)	32
2020 (35)	85	43	72	97	9,996 (6.3)	10
2021 (35)	84	31	75	97	682 (0.6)	0.7
**Eastern Mediterranean**						
2000 (21)	71	57	27	14	38,592 (4.5)	87
2010 (21)	76	62	52	38	10,072 (2.9)	17
2016 (21)	82	57	73	52	6,275 (4.7)	10
2019 (21)	83	52	76	38	18,458 (2.1)	26
2020 (21)	83	48	77	48	6,769 (4.3)	10
2021 (21)	82	48	77	52	26,089 (21.0)	40
**European**						
2000 (52)	91	60	48	38	37,421 (4.4)	50
2010 (53)	94	83	80	68	30,625 (8.9)	34
2016 (53)	93	81	88	77	4,440 (3.4)	5
2019 (53)	96	85	92	30	106,130 (12.2)	117
2020 (53)	94	77	91	70	10,945 (6.9)	14
2021 (53)	94	70	91	91	99 (0.1)	0.1
**South-East Asia**						
2000 (10)	62	27	3	0	78,558 (9.2)	51
2010 (11)	83	45	15	36	54,228 (15.8)	30
2016 (11)	89	64	75	27	27,530 (20.8)	14
2019 (11)	94	73	83	27	29,389 (3.4)	15
2020 (11)	88	64	80	45	9,389 (5.9)	5
2021 (11)	86	55	78	55	6,448 (5.2)	3
**Western Pacific**						
2000 (27)	85	48	2	26	177,052 (20.7)	106
2010 (27)	97	63	87	63	49,460 (14.4)	28
2016 (27)	96	63	93	48	57,879 (43.7)	31
2019 (27)	95	70	93	41	78,479 (9.0)	41
2020 (27)	94	63	94	37	6,605 (4.2)	4
2021 (27)	91	59	91	48	1,061 (0.9)	0.6

**Abbreviations**: MCV1 = first dose of measles-containing vaccine; MCV2 = second dose of measles-containing vaccine; WHO = World Health Organization.

* https://immunizationdata.who.int/pages/coverage/mcv.html (Accessed September 29, 2022).

^†^ Denominator is the number of WHO member states.

^§^ https://immunizationdata.who.int/pages/incidence/measles.html (Accessed September 29, 2022).

^¶^ Population data from United Nations Department of Economic and Social Affairs, Population Division, 2022. Any country not reporting measles cases for that year was removed from both the numerator and denominator in calculating incidence.

** Cases per 1 million population.

Among 194 WHO member states, 91 (47%) achieved ≥90% MCV1 coverage in 2021; however, among these countries, only 24 (26%) reported MCV1 coverage of ≥80% in all districts. In 2021, 24.7 million infants did not receive MCV1 through routine immunization services, an increase of 2.4 million (11%) from 2020. The 10 countries with the highest number of infants who did not receive MCV1 were Nigeria (3.1 million), India (2.5 million), Democratic Republic of the Congo (1.7 million), Ethiopia (1.7 million), Indonesia (1.2 million), Pakistan (1.2 million), Philippines (1.0 million), Angola (0.8 million), Brazil (0.7 million), and Tanzania (0.5 million). These countries accounted for 59% of all children who did not receive MCV1. Estimated MCV2 coverage quadrupled from 17% in 2000 to 72% in 2020, then declined slightly, to 71% in 2021. The number of countries offering MCV2 increased by 92%, from 95 (50%) in 2000 to 182 (94%) in 2021. Three countries (Comoros, Côte d’Ivoire, and Equatorial Guinea) introduced MCV2 in 2021.^††^

Approximately 150 million persons received MCV during supplementary immunization activities (SIAs)^§§^ in 18 countries in 2021. An additional 4 million persons received MCV during measles outbreak response activities. As of December 2021, 25 MCV campaigns that had been postponed since the start of the COVID-19 pandemic had been conducted; however, 18 MCV campaigns planned since March 2020 had still not been conducted, which resulted in an estimated 61 million postponed or missed MCV doses.

## Surveillance Performance and Reported Measles Incidence

WHO’s Global Measles and Rubella Laboratory Network (GMRLN) supports countries in providing standardized quality-controlled laboratory testing for measles and rubella. Among the 135 (70%) countries that reported discarded cases, 47 (35%) achieved the sensitivity indicator target of two or more discarded cases per 100,000 population in 2021, compared with 45 (31%) of 143 countries reporting in 2020. In 2021, GMRLN laboratories received 122,735 specimens for measles testing compared with 122,116 specimens in 2020.

Countries report the number of incident measles cases to WHO and UNICEF annually, using the Joint Reporting Form.^¶¶^ During 2000–2016, the number of reported measles cases decreased by 84%, from 853,479 to 132,490. Reported measles cases peaked at 873,022 in 2019, then declined to 159,073 in 2020, and 123,981 in 2021. From 2000 to 2016, annual measles incidence decreased 88%, from 145 cases per 1 million population to 18; measles incidence then increased to 120 cases per million in 2019 and decreased 82% to 21 in 2020 and 22% to 17 in 2021. In 2021, 22 countries in two WHO regions were affected by large and disruptive outbreaks***; 18 (82%) outbreaks occurred in countries in the African Region and four (18%) in the Eastern Mediterranean Region (Supplementary Table, https://stacks.cdc.gov/view/cdc/122049).

Genotypes detected from measles cases^†††^ were reported by 27 (33%) of the 82 countries reporting at least one measles case in 2021, compared with 45 (39%) of 115 countries reporting at least one measles case in 2020. The number of genotypes detected decreased from 13 in 2002 to six in 2014, three in 2020, and two in 2021. A total of 1,615 sequences were reported in 2020; among 648 reported sequences in 2021, 221 (34%) were D8 and 426 (66%) were B3.

## Measles Case and Mortality Estimates

A previously described model (*3*) for estimating measles cases and deaths was updated with 2021 measles data and United Nations 2000–2021 population estimates.^§§§^ Data on case fatality from an updated systematic review and a suite of covariates with known relationships to case fatality were used in a Bayesian meta-regression modeling framework to produce estimates of measles case fatality ratios^¶¶¶^ (*4*). The updated estimates reflect heterogeneity among countries, years, and ages. On the basis of the revised model and 2021 data, the estimated number of measles cases decreased 72%, from 34,013,000 in 2000 to 9,484,000 in 2021; estimated annual measles deaths decreased 83%, from 761,000 to 128,000 (Table 2). However, the estimated numbers of both cases and deaths were higher in 2021 compared with those in 2020. During 2000–2021, compared with no measles vaccination, measles vaccination prevented an estimated 56 million deaths globally (Figure).

**TABLE 2:** Estimated number of measles cases and deaths,* by World Health Organization region — worldwide, 2000 and 2021

Yr/WHO region (no. of countries in region)	No. (95% CI)		Estimated reduction in measles mortality, % 2000–2021	Cumulative no. of measles deaths averted by vaccination 2000–2021
	Estimated no. of measles cases	Estimated no. of measles deaths		
**Total, all regions**				
**2000 (191)**	**34,012,634 (27,393,416–42,901,683)**	**761,038 (561,895–993,312)**	**83**	**55,812,741**
**2021 (194)**	**9,484,464 (5,681,867–14,881,517)**	**127,656 (74,444–197,500)**		
**African**				
2000 (46)	10,965,152 (7,134,948–14,649,839)	356,299 (227,304–488,539)	81	19,499,793
2021 (47)	4,430,595 (2,619,358–7,030,815)	66,229 (38,811–106,293)		
**Americas**				
2000 (35)	8,770 (4,385–35,080)	NA^†^	61	4,343,128
2021 (35)	3,410 (1,705–13,640)	NA^†^		
**Eastern Mediterranean**				
2000 (21)	4,370,190 (3,295,576–6,213,952)	150,299 (115,962–205,857)	67	9,553,930
2021 (21)	2,303,170 (1,304,581–3,075,854)	48,979 (26,638–72,045)		
**European**				
2000 (52)	911,710 (733,732–1,353,344)	3,869 (3,098–4,747)	97	1,146,475
2021 (53)	86,196 (25,252–208,956)	132 (34–352)		
**South-East Asia**				
2000 (10)	12,395,953 (11,108,235–14,268,354)	224,822 (192,623–266,165)	95	16,546,297
2021 (11)	1,702,699 (1,399,591–2,321,389)	10,230 (8,328–13,538)		
**Western Pacific**				
2000 (27)	5,360,859 (5,116,541–6,381,114)	25,746 (22,907–27,993)	92	4,723,119
2021 (27)	958,395 (331381–2,230,863)	2,085 (633–5,267)		

**Abbreviations:** NA = not applicable; WHO = World Health Organization.

* The measles mortality model used to generate estimated measles cases and deaths is rerun each year using the new and revised annual WHO-UNICEF estimates of national immunization coverage data as well as updated surveillance data. In 2021, data on case fatality from an updated systematic review and a suite of covariates with known relationships to case fatality were used in a Bayesian meta-regression modeling framework to produce estimates of measles case fatality ratios; therefore, the estimated number of cases and mortality estimates for 2000–2020 in this report differs from previous reports.

^†^ Estimated measles mortality was too low to allow reliable measurement of mortality reduction.

**FIGURE:**
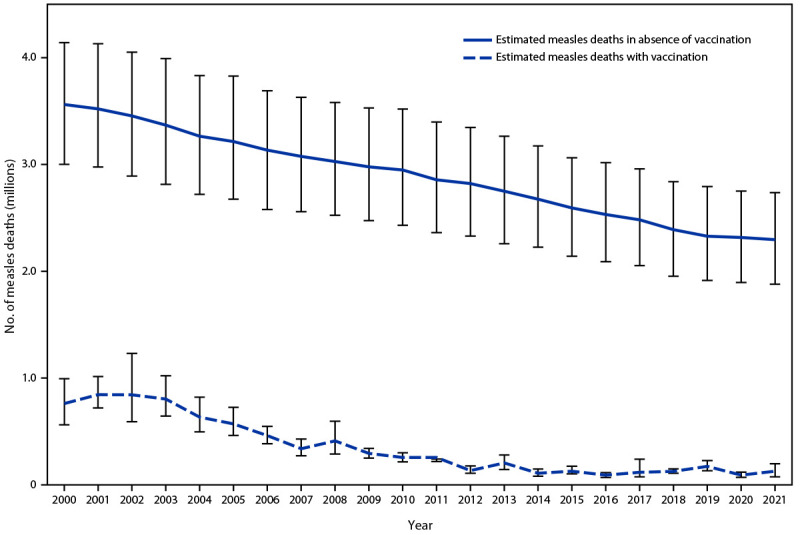
Estimated number of annual measles deaths with measles vaccination and in the absence of measles vaccination — worldwide, 2000–2021* * Deaths prevented by vaccination are estimated by the area between estimated deaths with vaccination and those without vaccination (cumulative total of 56 million deaths prevented during 2000–2021). Vertical bars represent 95% CIs around the point estimate.

## Regional Verification of Measles Elimination

By the end of 2021, 76 (39%) countries had been verified by independent regional commissions as having achieved or maintained measles elimination status. No WHO region had achieved and sustained elimination, and no African Region country has yet been verified to have eliminated measles. WHO’s Region of the Americas achieved verification of measles elimination in 2016; however, endemic measles transmission was reestablished in Venezuela (2016) and Brazil (2018). Since 2016, endemic transmission has been reestablished in eight other countries (Albania, Cambodia, Lithuania, Mongolia, Slovakia, the Czech Republic, the United Kingdom, and Uzbekistan) that had previously achieved verification of measles elimination.

## Discussion

All WHO regions remain committed to measles elimination; however, no region has achieved and sustained elimination targets. Drops in MCV1 coverage and declines in surveillance performance that started or continued during the COVID-19 pandemic persisted in 2021 (*5*–*7*). Among regions, the Southeast Asia Region faced the largest decrease in MCV1 coverage (from 94% to 86%) between 2019 and 2021, and only the European Region maintained MCV1 coverage from 2020 to 2021. None of the WHO regions have recovered MCV1 or MCV2 coverage levels from 2019, which were still below the 95% coverage needed to achieve and sustain measles elimination (*8*).

SIAs represent an opportunity to reach children with missing MCV doses from the routine immunization program and close immunity gaps.**** In 2021, the implementation of 25 campaigns that had been delayed because of COVID-19 indicates some pandemic recovery; however, 18 pending SIAs that had not yet been conducted as of December 2021 present a risk for measles outbreaks.

The observed decrease in measles incidence in 2020 and 2021 could reflect actual changes related to increased immunity after a 2017–2019 global resurgence of measles, reduced viral transmission associated with COVID-19 mitigation measures, limited detection resulting from surveillance system underperformance, or a combination of multiple factors (*1*,*2*,*9*). Sensitivity of measles surveillance remained low in 2021, with continued low numbers of specimens received for laboratory testing and few countries achieving the surveillance sensitivity indicator. Sustained declines in surveillance not only affect the timely detection of cases and outbreaks but also undermine a program’s ability to use measles as a tracer to highlight gaps in the overall immunization system.

The findings in this report are subject to at least three limitations. First, not all countries report complete, or any, data for SIAs and outbreak response activities; therefore, the numbers on these activities provided in this report could be underestimated. Second, the measles estimation model was updated this year, which limits comparability with estimates from previous years. Finally, the number of specimens submitted for genotyping represents a fraction of measles cases; hence, data presented in this report might not reflect the actual global distribution of genotypes.

Declining routine MCV coverage and delays in SIAs in 2021 left millions of children with zero or only 1 dose of MCV. In the absence of a high-performing surveillance system to promptly detect cases, a growing population of susceptible children is at risk for measles disease and outbreaks. In alignment with IA2030, the Measles and Rubella Strategic Framework 2021–2030^††††^ outlines strategies for countries to build robust, case-based surveillance for measles to detect immunity gaps and outbreaks, identify root causes of undervaccination, and develop targeted solutions, including catch-up immunization for those who missed routine immunization doses during the pandemic, to reach all children with 2 doses of MCV. Accelerating these measures will help regain historical progress toward regional measles elimination.

**Summary**

**What is already known about this topic?**

Progress toward measles elimination experienced setbacks in 2020 during the COVID-19 pandemic.

**What is added by this report?**

During 2000–2021, measles vaccination prevented an estimated 56 million deaths worldwide. In 2021, only 81% of children received their first dose of measles containing vaccine (MCV), the lowest coverage reported since 2008, leaving 25 million children vulnerable to measles. Measles surveillance continues to be suboptimal, and large and disruptive outbreaks were reported in 22 countries.

**What are the implications for public health practice?**

Reaching all children with 2 doses of MCV and strengthening measles surveillance is critical to close immunity gaps, prevent outbreaks, and recover progress lost during the pandemic.

## Footnotes

* Measles elimination is defined as the absence of endemic measles virus transmission in a region or other defined geographic area for ≥12 months in the presence of a high-quality surveillance system that meets the targets of key performance indicators.

†
https://www.who.int/teams/immunization-vaccines-and-biologicals/strategies/ia2030

§ A discarded measles case is defined as a suspected case that has been investigated and determined to be neither measles nor rubella by using either 1) laboratory testing in a proficient laboratory, or 2) epidemiologic linkage to a laboratory-confirmed outbreak of a communicable disease that is not measles or rubella. The discarded case rate is used to measure the sensitivity of measles surveillance.

¶ Estimates based on administrative data as well as any other available information on factors affecting immunization coverage, including private or nongovernmental organization sector contributions to immunization, difficulties with demographic data, and incomplete reporting.

** Calculated for MCV1, among children aged 1 year or, if MCV1 is administered at age ≥1 year, among children aged 24 months. Calculated for MCV2 among children at the recommended age for the administration of MCV2 per the national immunization schedule. https://www.ncbi.nlm.nih.gov/pmc/articles/PMC8464208/

†† Data as of September 20, 2022. http://immunizationdata.who.int/pages/vaccine-intro-by-antigen/mcv2.html?ISO_3_CODE=&YEAR=

§§ SIAs are typically carried out using two target age ranges: 1) an initial catch-up SIA focuses on infants, children, and adolescents aged 9 months–14 years to eliminate susceptibility to measles in a population, and 2) periodic follow-up SIAs then focus on infants and children aged 9–59 months born since the last SIA. Follow-up SIAs typically are conducted nationwide every 2–4 years to eliminate measles susceptibility in recent birth cohorts because of low MCV coverage and to protect infants, children, and adolescents who did not respond to MCV1. Countries can provide additional data to WHO, and data are updated retrospectively. https://immunizationdata.who.int/listing.html?topic=&location=

¶¶ Only countries that reported data are in the numerator and denominator. Countries do not provide WHO with their reasons for not reporting measles cases. https://immunizationdata.who.int/pages/incidence/measles.html (Accessed September 20, 2022).

*** The IA2030 global monitoring framework defines large and disruptive outbreaks as having at least 20 cases per 1 million population.

††† Data as of September 20, 2022. https://who-gmrln.org/means2

§§§ State-space model of unobserved measles incidence during 2000–2021 generated using the following inputs from all member countries: 1) total annual reported measles cases, 2) annual MCV1 coverage from WHO-UNICEF estimates of national immunization coverage, 3) annual MCV2 coverage from WHO-UNICEF estimates of national immunization coverage, 4) annual SIAs with coverage and age targets (subnational SIAs are discounted by the proportion of the total population targeted), 5) annual total population size, 6) total annual births, and 7) list of all countries and years for which reporting was enhanced.

¶¶¶ The model fitted the reported case fatality ratios from the systematic review as a function of the following covariates: 1) gross domestic product per capita, 2) HIV prevalence, 3) maternal education, 4) MCV1 coverage, 5) proportion urban, 6) total fertility rate, 7) mortality rate among children aged <5 years, 8) vitamin A deficiency prevalence, 9) war and terrorism mortality rate, 10) wasting prevalence, and 11) measles incidence. Annual measles incidence for each country was based on this fitted state-space model. High income countries were excluded from this analysis.

**** https://apps.who.int/iris/handle/10665/340657; https://apps.who.int/iris/handle/10665/360891

††††
https://www.who.int/publications/i/item/measles-and-rubella-strategic-framework-2021-2030
